# Osimertinib in poor performance status patients with T790M-positive advanced non-small-cell lung cancer after progression of first- and second-generation EGFR-TKI treatments (NEJ032B)

**DOI:** 10.1007/s10147-021-02043-2

**Published:** 2021-10-13

**Authors:** Yukari Tsubata, Kana Watanabe, Ryota Saito, Atsushi Nakamura, Hiroshige Yoshioka, Mami Morita, Ryoichi Honda, Nobuhiro Kanaji, Satoshi Ohizumi, Daisuke Jingu, Taku Nakagawa, Kensuke Nakazawa, Atsuto Mouri, Susumu Takeuchi, Naoki Furuya, Yuki Akazawa, Kiyotaka Miura, Eiki Ichihara, Makoto Maemondo, Satoshi Morita, Kunihiko Kobayashi, Takeshi Isobe

**Affiliations:** 1 Department of Internal Medicine, Division of Medical Oncology and Respiratory Medicine, Shimane University Faculty of Medicine, 89-1 Enya-cho, Izumo, Shimane 693-8501 Japan; 2 Department of Respiratory Medicine, Miyagi Cancer Center, 47-1, Nodayama, Medeshimashiote, Natori, Miyagi 981-1293 Japan; 3 Department of Respiratory Medicine, Tohoku University Hospital, 1-1, Seiryo-machi, Aoba-ku, Sendai, Miyagi 980-8574 Japan; 4 Department of Pulmonary Medicine, Sendai Kousei Hospital, 4-15, Hirose-machi, Aoba-ku, Sendai, Miyagi 980-0873 Japan; 5 Department of Thoracic Oncology, Kansai Medical University Hospital, 2-3-1, Shin-machi, Hirakata, Osaka 573-1191 Japan; 6 Department of Respiratory Medicine, Asahi General Hospital, Asahi, Chiba 1326289-2511 Japan; 7 Department of Internal Medicine, Division of Hematology, Rheumatology and Respiratory Medicine, Faculty of Medicine, Kagawa University, 1750-1, Ikenobe, Miki-cho, Kida-gun, Kagawa 761-0793 Japan; 8 Department of Respiratory Medicine, National Hospital Organization Hokkaido Cancer Center, 2-3-54, 4-jyo, Kikusui, Shiraisi-ku, Sapporo, Hokkaido 003-0804 Japan; 9 Department of Respiratory Medicine, Saka General Hospital, 16-5, Nishiki-cho, Shiogama, Miyagi 985-8506 Japan; 10 Department of Thoracic Surgery, Omagari Kosei Medical Center, 8-65, Toori-machi, Omagari, Daisen, Akita 014-0027 Japan; 11 Division of Clinical Medicine, Department of Pulmonary Medicine, Faculty of Medicine, University of Tsukuba, 2-1-1, Amakubo, Tsukuba, Ibaraki 305-8576 Japan; 12 Department of Respiratory Medicine, Saitama Medical University International Medical Center, 1397-1, Yamane, Hidaka, Saitama 350-1298 Japan; 13 Division of General Thoracic and Thyroid Surgery, Tokyo Medical University, 6-7-1, Nishi-shinjuku, Shinjuku, Tokyo, 160-0023 Japan; 14 Division of Respiratory Medicine, Department of Internal Medicine, St. Marianna University School of Medicine, 2-16-1, Sugao, Miyamae-ku, Kawasaki, Kanagawa 216-8511 Japan; 15 Department of Thoracic Oncology, National Hospital Organization Osaka Toneyama Medical Center, 5-1-1, Toneyama, Toyonaka, Osaka 560-8552 Japan; 16 Department of Respiratory Medicine, Shimane Prefectural Central Hospital, 4-1-1, Himehara, Izumo, Shimane, 693-8555 Japan; 17 Department of Allergy and Respiratory Medicine, Okayama University Hospital, 2-5-1, Shikata-cho, Kita-ku, Okayama, 700-8558 Japan; 18 Division of Pulmonary Medicine, Department of Internal Medicine, Iwate Medical University School of Medicine, 2-1-1, Idaidoori, Yahaba-cho, Shiwa-gun, Iwate, 028-3695 Japan; 19 Department of Biomedical Statistics and Bioinformatics, Kyoto University Graduate School of Medicine, 54, Shogoinkawahara-cho, Sakyo-ku, Kyoto, 606-8507 Japan

**Keywords:** EGFR T790M, Non-small-cell lung cancer, Osimertinib, Phase II, Poor performance status

## Abstract

**Background:**

Osimertinib is effective in patients with T790M mutation-positive advanced non-small-cell lung cancer (NSCLC) resistant to epidermal growth factor receptor (EGFR) tyrosine kinase inhibitors (TKIs). However, its effectiveness and safety in patients with poor performance status (PS) are unknown.

**Methods:**

Enrolled patients showed disease progression after treatment with gefitinib, erlotinib, or afatinib; T790M mutation; stage IIIB, IV, or recurrent disease; and PS of 2–4. Osimertinib was orally administered at a dose of 80 mg/day. The primary endpoint of this phase II study (registration, jRCTs061180018) was response rate and the secondary endpoints were progression-free survival (PFS), overall survival (OS), disease control rate, and safety.

**Results:**

Thirty-three patients were enrolled, of which 69.7% and 24.2% had PS of 2 and 3, respectively. One patient was excluded due to protocol violation; in the remaining 32 patients, the response rate was 53.1%; disease control rate was 75.0%; PFS was 5.1 months; and OS was 10.0 months. The most frequent adverse event of grade 3 or higher severity was lymphopenia (12.1%). Interstitial lung disease (ILD) was observed at all grades and at grades 3–5 in 15.2% (5/33) and 6.1% (2/33) of patients, respectively. Treatment-related death due to ILD occurred in one patient. Patients negative for activating EGFR mutations after osimertinib administration had longer median PFS than those positive for these mutations.

**Conclusion:**

Osimertinib was sufficiently effective in EGFR-TKI-resistant, poor PS patients with T790M mutation-positive advanced NSCLC. Plasma EGFR mutation clearance after TKI treatment could predict the response to EGFR-TKIs.

## Introduction

Epidermal growth factor receptor (*EGFR*) gene mutations are the most common driver oncogene mutations associated with non-small cell lung cancer (NSCLC), accounting for 55% of driver oncogene mutations in lung adenocarcinoma cases in East Asia [1]. The recommended treatments for stage IV EGFR-positive lung cancer are EGFR-tyrosine kinase inhibitor (TKI) monotherapy, EGFR-TKI plus cytotoxic combination chemotherapy, and EGFR-TKI plus anti-angiogenic combination therapy [2]. A promising response rate (RR) and prolongation of progression-free survival (PFS) have been reported for each of these treatments [3–7]. Nonetheless, disease progression is observed after 9–21 months in almost all patients who respond to treatment [3–7]. The EGFR T790M mutation is considered a cause of acquired resistance to EGFR-TKI therapy and is found in approximately 60% of patients with lung adenocarcinoma treated with EGFR-TKIs [8, 9].

Osimertinib is a third-generation EGFR-TKI. A clinical trial comparing osimertinib with pemetrexed plus either carboplatin or cisplatin in patients with EGFR T790M mutation-positive NSCLC and with disease progression after first-line therapy reported significantly longer PFS (10.1 months vs. 4.4 months) and significantly better response rates (71% vs. 31%) with osimertinib [10]. Favorable outcomes have also been achieved with osimertinib, as the first-line therapy in patients with EGFR-positive stage IV NSCLC [11]. Notably, osimertinib has been shown to be effective in patients with central nervous system (CNS) metastasis based on a subgroup analysis of such patients [12]. Therefore, osimertinib is a key drug for EGFR-positive patients with CNS metastases. Osimertinib is used as a standard therapy in patients with performance status (PS) scores of 0–1 [2]. In addition, specific TKI therapy for the driver oncogene mutation is recommended for patients with mutation-positive lung cancer but a PS score of 2 due to the demonstrated efficacy in patients with good PS and the likelihood of a good response [2]. For patients with PS scores of 3–4, best supportive care is indicated, and aggressive anticancer treatment is not recommended. A first-generation EGFR-TKI, gefitinib, was efficacious in patients with EGFR-positive lung cancer and poor PS [13], but the clinical utility and safety of osimertinib, a third-generation EGFR-TKI, remain unclear.

Therefore, we conducted an open-label, multicenter, single-arm phase II study to evaluate the effectiveness and safety of osimertinib in patients with EGFR T790M mutation-positive advanced NSCLC with Eastern Cooperative Oncology Group (ECOG) PS scores of 2–4.

## Patients and methods

### Patients

The main eligibility criteria were as follows:

- Non-radiocurable stage IIIB, IIIC, IVA, or IVB, or postoperative recurrent NSCLC confirmed either histologically or cytologically
- Positive for an EGFR-sensitizing mutation (G719X, exon 19 deletion, exon 21 L858R point mutation, or exon 21 L861Q point mutation)
- Imaging-confirmed disease progression after treatment with a first- or second-generation EGFR-TKI (gefitinib, erlotinib, or afatinib)
- EGFR T790M mutation confirmed in a specimen collected after disease progression following the most recent treatment regimen (all methods used to determine the EGFR T790M mutation status were accepted)
- Aged 20 years or older at the time of informed consent
- ECOG PS score of 2–4, with the performance decline determined to be due to lung cancer by the attending physician

Patients who received multiple EGFR-TKIs or used vascular endothelial growth factor inhibitors or cytotoxic chemotherapy in combination with an EGFR-TKI were also eligible.

The main exclusion criteria were as follows:

- History of interstitial lung disease (ILD), drug-induced ILD, radiation pneumonitis requiring steroid therapy, or evidence of an active ILD
- Previous immune checkpoint inhibitor treatment
- Clinically unstable brain metastasis
- Abnormal electrocardiogram, prolonged QTc, or a factor increasing the risk of induced arrhythmia

### Study design and treatment

NEJ032B was a multicenter, single-arm, phase 2 study assessing the efficacy and safety of osimertinib in patients with EGFR T790M-positive NSCLC. The study was conducted in 17 institutions across Japan from February 2017 to May 2019.

Patients with confirmed EGFR T790M-positive lung cancer received 80 mg of oral osimertinib once daily. Patients received the treatment until progressive disease (PD), unacceptable toxicity, or consent withdrawal. Until the fourth week after the start of treatment, the condition of patients and results of laboratory tests were examined weekly. CT imaging was performed at least every 8 weeks, and confirmation was performed 4 weeks later in patients in whom complete response (CR) or partial response (PR) was confirmed. The treatment efficacy was assessed using the Response Evaluation Criteria in Solid Tumors (RECIST), version 1.1. Adverse events were evaluated using the Common Terminology Criteria for Adverse Events (CTCAE), version 4.0. The efficacy and safety assessments were conducted by the Central Effectiveness and Safety Assessment Committee.

### Plasma sample collection and EGFR mutation analysis

The NEJ032B biomarker study was conducted in patients who consented to the biomarker study. The plasma ctDNA analysis to detect the activating EGFR mutations and T790M mutation was performed using an improved PNA-LNA Polymerase Chain Reaction (PCR) clamp method (LSI Medience Corporation, Tokyo, Japan). Whole blood samples (21 mL) were collected in ethylenediaminetetraacetic acid (EDTA) tubes before TKI treatment (P0), 8 weeks after the initiation of study treatment (P1), and after disease progression (P2). Samples were mixed thoroughly, and the plasma isolated by centrifuging blood at 2000×*g* for 10 min was stored at − 20 °C. DNA was extracted from plasma samples using the QIAamp Circulating Nucleic Acid kit. PCR primers were designed to amplify G719X, exon 19 deletion, T790M, L858R, and L861Q. LNA probes were prepared complementary to each mutant allele, and PNA clamps were complementary to the respective wild-type alleles.

### Statistical analysis

Statistical analyses were performed using Statistical Analysis System version 9.4 (SAS Institute Inc., North Carolina, USA). The primary endpoint was RR, and secondary endpoints were disease control rate, time to treatment failure, PFS, overall survival (OS), PS improvement, safety, and tolerability.

Osimertinib therapy has a reported RR of 63.6% in EGFR T790M-positive patients [14]. For the present study, an RR of 25% was considered a clinically meaningful threshold for patients with inoperable or recurrent NSCLC. Assuming a decrease of approximately 10% based on previous study results in patients with good PS, the anticipated RR was set at 50%. Given an α-error of 0.025 (one-sided) and β-error of 0.2, the required number of patients was determined to be 29. Based on this and allowing for dropouts, the target number of patients was set at 32. The most informative secondary endpoint to clinical status was PS improvement, which was defined as the proportion of per-protocol patients whose PS during osimertinib treatment was improved from baseline. PFS was defined as the interval between the months relapsed form the day of enrollment and the date of the first observation of disease progression or death from any cause. Patients who were alive without disease progression at the data cut-off point (May 21, 2020) were censored at the last point, as the patients were assessed to be progression-free. PFS and OS were estimated using the Kaplan–Meier method.

### Ethics

This study was conducted in accordance with the principles of the Declaration of Helsinki and the Good Clinical Practice Guidelines. All patients provided written informed consent. The study protocol conformed with the Clinical Trials Act of 2017, was approved by the certified clinical research review board of Shimane University, and is published on the Japan Registry of Clinical Trials (jRCTs061180018).

## Results

### Patient characteristics

Thirty-three patients were enrolled in the study between February 2017 and May 2019. The primary endpoint RR was calculated in a per-protocol set of 32 patients because one patient violated the protocol; the patient received a prohibited concomitant therapy (radiotherapy). All other endpoints, including safety, were analyzed in the full analysis set. The median age of the enrolled patients was 72 (47–89) years, and most patients were women (27 patients, 81.8%). The most common PS score was 2 (23 patients, 69.7%), and the most common previous treatment was EGFR-TKI monotherapy (18 patients, 54.5%) (Table 1).

**Table 1:** Patient demographic and clinical characteristics at baseline

Total (*N*)	33
Median age (range)	72 (47–89)
Sex (male/female) (%)	6/27 (18.2/81.8)
ECOG PS 2/3/4 (%)	23/8/2 (69.7/24.2/6.1)
Clinical stage before starting protocol treatment: IVA/IVB/postoperative recurrence (%)	7/23/3 (21.2/69.7/9.1)
Prior treatment	
EGFR-TKI alone	18 (54.5)
EGFR-TKI/cytotoxic anticancer agent/another molecularly targeted drug	7 (21.2)
EGFR-TKI/cytotoxic anticancer agent	6 (18.2)
EGFR-TKI/another molecularly targeted drug	2 (6.1)
Line of treatment: second/third/fourth/fifth or later (%)	16/6/3/8 (48.5/18.2/9.1/24.2)
Histopathological classification: adenocarcinoma (%)	33 (100)
Brain metastasis: present (%)	16 (48.5)

*ECOG PS* Eastern Cooperative Oncology Group performance status, *EGFR-TKI* epidermal growth factor receptor tyrosine kinase inhibitor

### Efficacy

**Fig. 1:**
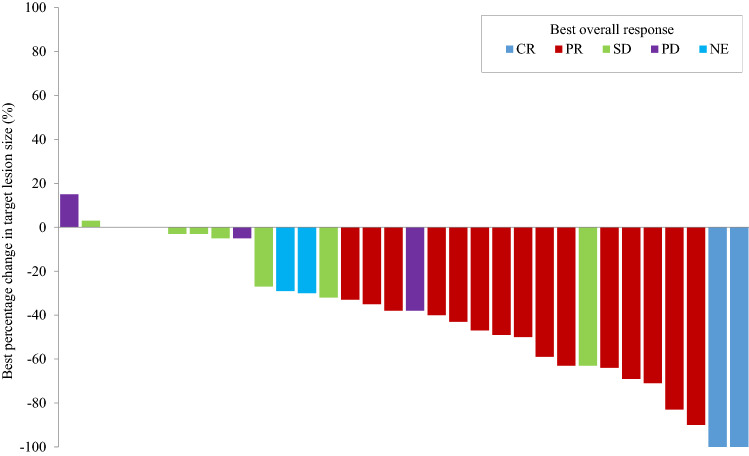
Waterfall plot of the best percentage change in target lesion size. *CR* complete response, *NE* non-evaluable, *PD* progressive disease, *PR* partial response, *SD* stable disease

In the per-protocol set of 32 patients, the primary endpoint RR was 53.1% (95% confidence interval [CI]: 34.7–70.9), which exceeded the preestablished criterion and therefore met the anticipated RR. The disease control rate was 75.0% (95% CI: 56.6–88.5) with two patients achieving a CR (Table 2, Fig. 1). In the subset analysis by PS, for PS 2 (23 patients), the RR was 60.9% and DCR was 82.6%. For PS 3–4 (10 patients), the RR was 30.0% and the DCR was 50.0%.

**Table 2:** Response rate and disease control rate

Total (*N*)	32
Complete response (%)	2 (6.3)
Partial response (%)	15 (46.9)
Stable disease (%)	7 (21.9)
Progressive disease (%)	5 (15.6)
Non-evaluable (%)	3 (9.4)
Response rate (%, 95% CI)	17 (53.1, 34.7–70.9)
Disease control rate (%, 95% CI)	24 (75.0, 56.6–88.5)

*CI* confidence interval

**Fig. 2:**
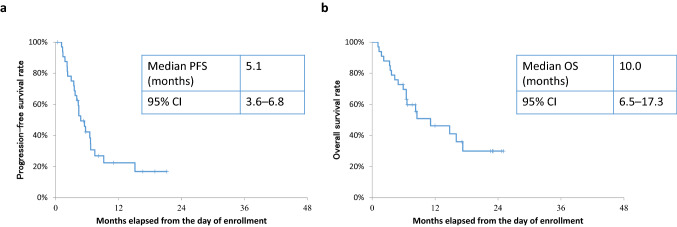
Kaplan–Meier curves for progression-free survival (PFS) and overall survival (OS). **a** PFS, median observation period: 4.8 months, events occurred in 25/33 patients. **b** OS, median observation period: 10.0 months, events occurred in 24/33 patients. CI, confidence interval

**Fig. 3:**
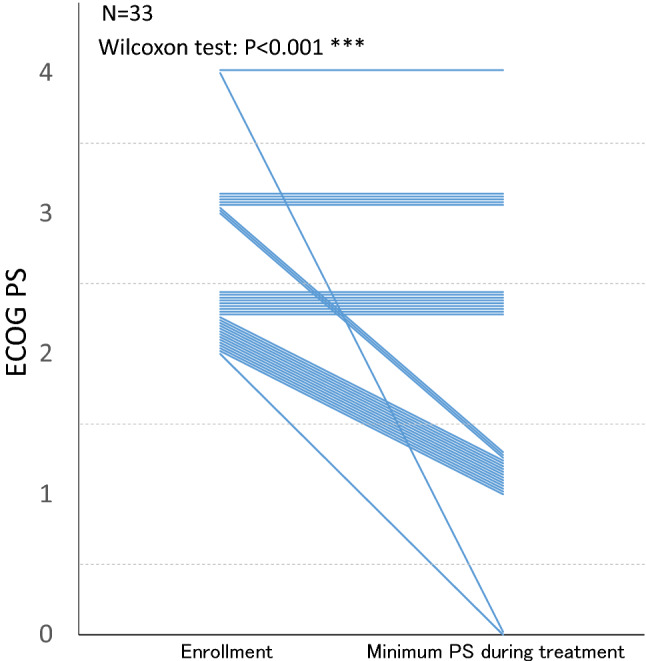
Change in the performance status of each patient during treatment. Each line shows the change in performance status (PS) of a patient from baseline to their best status during the treatment (lowest observed PS from the day of the first dose to the day treatment was stopped). A clinically significant improvement was observed in 54.5% (95% CI 36.4–71.9) of patients. ECOG PS, Eastern Cooperative Oncology Group performance status

The median PFS was 5.1 months (95% CI: 3.55–6.67), the median OS was 10.0 months (95% CI: 6.51–17.3) (Fig. 2), and the time to treatment failure was 4.3 months (95% CI: 2.96–5.49). In the subset analysis by PS, for PS 2, the median PFS and OS were 6.5 and 17.5 months, respectively. For PS 3–4, the median PFS and OS were 3.0 and 4.8 months, respectively. The PS improvement rate was 54.5% (95% CI: 36.4–71.9, *P* < 0.001), which is a good result (Fig. 3). A comparison of the smallest PS score during protocol treatment with the baseline PS score revealed no increase in PS in any patient.

### Plasma EGFR mutations

**Fig. 4:**
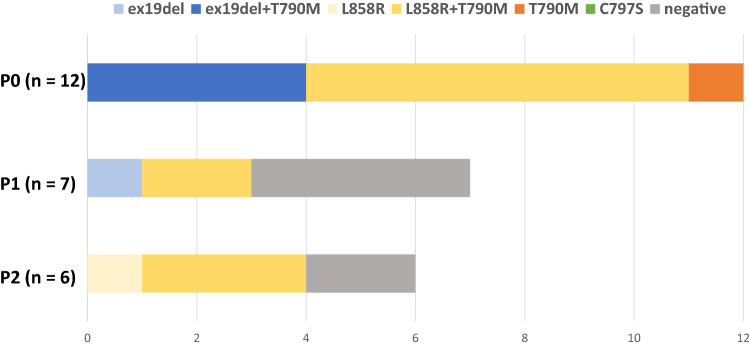
EGFR mutation status at different time points of osimertinib treatment. This figure depicts the percentage of each mutation at each time point of osimertinib treatment (P0, P1, and P2). The horizontal axis shows the number of patients. P0: plasma samples before the start of the study treatment, P1: plasma samples 8 weeks after the start of the study treatment, P2: plasma samples after disease progression

**Fig. 5:**
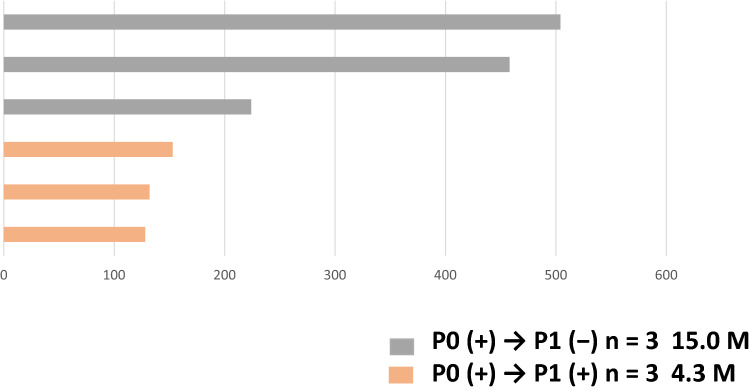
Swimmer plot of progression-free survival (PFS) between patients with clearance and non-clearance of the activating EGFR mutations**.** Each histogram shows PFS. Gray histograms show the PFS of patients who experienced plasma clearance of activating mutations. Orange histograms show the PFS of patients with sustained plasma-activating mutations. P0: plasma samples before the start of the study treatment, P1: plasma samples 8 weeks after the start of the study treatment. (+): positive for plasma EGFR mutations, (−): negative for plasma EGFR mutations

The total number of collected plasma samples was 12 (37.5%) at P0, 7 (21.9%) at P1, and 6 (18.8%) at P2 (Table 3). The frequencies of plasma-activating EGFR mutations and drug-resistant mutation (T790M) before the study treatment (P0) were 91.7% and 100%, respectively. There was one case in which only T790M was detected without the activating EGFR mutations. Of the six cases positive for plasma-activating EGFR mutations at P0, three had no plasma mutations at P1 and three were still positive at P1 (Fig. 4). Among patients with detectable activating EGFR mutations at P0, the median PFS was longer for those in whom activating mutations were not detected at P1 than for those in whom the mutations were still detectable at P1 (15.0 vs. 4.3 months, respectively) (Fig. 5). Of the six cases positive for T790M mutation at P0, four had no T790M mutations at P1 and two were still positive at P1. The median PFS of T790M-negative cases at P1 was 11.2 months and that of the T790M-positive cases at P1 was 4.6 months (data not shown).

**Table 3:** Detection of EGFR mutations in plasma samples

	Pre-treatment P0 (%)	Under-treatment P1 (%)	Post-PD P2 (%)
Number of samples	12	7	6
Activating mutation	11 (91.7)	3 (42.9)	4 (66.7)
Drug-resistant mutation (T790M)	12 (100)	2 (28.6)	3 (50.0)

### Safety

The most frequent adverse event of any grade was anemia (81.8%), although anemia of grade 3 or greater severity occurred in just 6.1% of patients (Table 4). The most frequent adverse event of grade 3 or greater severity was lymphopenia at 12.1%. ILD was reported in five cases (15.2%), of which two were grade 3 or higher; one of these was a treatment-related death.

**Table 4:** Adverse events

Adverse event	All grades (%)	Grade 3 or greater severity (%)
Anemia	27 (81.8)	2 (6.1)
Hypoalbuminemia	22 (66.7)	2 (6.1)
Hyponatremia	18 (54.5)	2 (6.1)
Hypocalcemia	15 (45.5)	1 (3.0)
Increased ALP	15 (45.5)	1 (3.0)
Thrombopenia	13 (39.4)	0
Lymphopenia	12 (36.4)	4 (12.1)
Increased AST	12 (36.4)	2 (6.1)
Leukopenia	11 (33.3)	0
Increased ALT	9 (27.3)	2 (6.1)
Hyperkalemia	9 (27.3)	1 (3.0)
Increased creatinine	9 (27.3)	0
Proteinuria	8 (24.2)	0
Nail disorder	7 (21.2)	0
Hypertension	6 (18.2)	1 (3.0)
Rash	5 (15.2)	1 (3.0)
Acne-like eruption	5 (15.2)	1 (3.0)
Xerosis cutis	5 (15.2)	0
Anorexia	5 (15.2)	3 (9.1)
Interstitial lung disease	5 (15.2)	2 (6.1); G5 in 1 patient
Hypermagnesemia	4 (12.1)	1 (3.0)
Hypokalemia	4 (12.1)	0
Diarrhea	4 (12.1)	0

*ALP* alkaline phosphatase, *ALT* alanine transaminase, *AST* aspartate transaminase

Dose reduction due to toxicity occurred in five patients (15.2%). The reasons for this were increased aspartate transaminase and alanine transaminase levels, acne-like eruption, oral mucositis, purpura, urinary tract infection, and anorexia. Treatment was stopped in seven patients due to toxicity (21.1%); the reasons were ILD, prolonged QT, corneal ulcer, inability to restart therapy after drug cessation, and discretion of the attending physician due to safety concerns.

## Discussion

A few clinical studies have examined EGFR-TKI therapy in patients with EGFR-positive lung cancer and poor PS. Here, we confirmed that osimertinib is both efficacious and safe, which is beneficial for routine medical practice and therapeutic options for patients with T790M mutation-positive advanced NSCLC with EGFR-TKI resistance and poor PS, particularly for T790M-positive patients with no other treatment options.

This was a phase II investigator-initiated clinical trial conducted in patients who were T790M positive with a PS score of 2–4 and at least one failed EGFR-TKI treatment regimen. Although the median age of the participants was high at 72 years, and approximately 50% of the participants received at least their third-line therapy in this study, the RR of 53.1% was extremely good. The efficacy of osimertinib has previously been demonstrated in two clinical trials in patients with EGFR-positive lung cancer and good PS, where the T790M variant was confirmed after treatment—the phase III AURA3 study that compared osimertinib with platinum-doublet chemotherapy (median age: 62 years) [10] and a pooled analysis of the phase II AURA and AURA2 studies (median age: 62 years) [14, 15]. In these clinical studies, the RR with osimertinib was 71% and 66%, respectively. Meanwhile, Nakashima et al. reported a 53% RR in prospective phase II study of poor PS [16]. The RR in our study not only met the study endpoint statistically but was also excellent considering that this study was conducted in older patients with poor PS. In the above two studies on good PS, the PFS with osimertinib was 10.1 and 9.9 months, respectively, which is substantially different from the 5.1 months observed in the present study. Among clinical studies that used the first-generation EGFR-TKI gefitinib, one study of first-line therapies in patients with EGFR-positive lung cancer and good PS reported a PFS of 10.8 months [3], whereas another conducted in patients with poor PS reported a shorter PFS of 6.5 months [13]. Based on this, the different PS scores of patients enrolled in our study and other similar studies may be a major factor in their different clinical outcomes. We also suspect that clinical outcomes were affected by the differences in patient characteristics, such as older age, longer treatment history, and higher frequency of brain metastasis in our study.

In this study, we observed a PS improvement rate of 54.5% and PS scores that either remained the same or temporarily improved during treatment. We also observed a potential “Lazarus effect” in one patient whose PS score improved from 4 to 0. Osimertinib is currently used as a first-line therapy for EGFR-positive cancer, because it is well indicated for cases that are EGFR-TKI-resistant and T790M-positive, even when the patient has poor PS; however, rebiopsy must be seriously considered in patients whose first EGFR-TKI therapy is not osimertinib.

The safety evaluation in this study revealed anemia, lymphopenia, leukopenia, and other signs of myelosuppression, although all events were mild in severity and acceptable. Adverse events encountered with first- and second-generation EGFR-TKI therapies were eruption, diarrhea, and impaired liver function, but impaired liver function of grade 3 or greater severity only occurred in 6.1% of patients in this study. Nonetheless, one-fifth of patients in this study developed toxicity that required cessation of the treatment.

We also observed a high overall incidence of drug-induced ILD at 15.2%, with one case of death. Although the pooled analysis of the AURA and AURA2 studies showed a 3% incidence of drug-induced ILD [15], an analysis of real-world data on osimertinib revealed that the incidence of drug-induced ILD was 6.8% [17]. Given these findings, the relatively high incidence of drug-induced ILD observed in this study was likely due to the poor patients’ PS, as well as ethnic differences, given that our study was conducted in Japanese patients. While there is no data on the efficacy and safety of osimertinib as the first-line therapy in patients with EGFR mutation-positive lung cancer and poor PS, based on the results of this study, caution should be paid to the possibility of drug-induced ILD when using osimertinib to treat patients with poor PS, even as a first-line therapy. Currently, osimertinib is used as a first-line therapy for EGFR-positive lung cancer.

Here, plasma-activating EGFR mutations and T790M mutation were detected at a high frequency using the PNA-LNA PCR clamp method before the study treatment. The high detection rate of EGFR mutations in this study might be related to the poor PS induced by high tumor burden in the patients. The plasma clearance of activating mutations during TKI treatment represents a potential predictive factor for response to TKI treatment [18–20].

Patients with EGFR-positive lung cancer are expected to respond to EGFR-TKI therapy, although almost all develop resistance 1–2 years after starting treatment. There are a variety of mechanisms of acquired resistance, with the T790M mutation reportedly accounting for 60% of patients who develop resistance [8]. Osimertinib can circumvent EGFR-TKI resistance, providing an excellent treatment option for patients in whom the T790M variant is confirmed after first- or second-generation EGFR-TKI therapy. This view is supported by the favorable response observed in this study, even among patients with poor PS. Our study was limited by the relatively small number of patients, and therefore, we could not conduct subgroup analysis. To enable the analysis of CNS reactions, we believe that it is necessary to focus on patients with CNS metastases in the future.

Osimertinib therapy exhibits acceptable efficacy in patients with T790M mutation-positive advanced NSCLC with EGFR-TKI resistance and poor PS; however, adverse events and ILD should be considered.

## Data Availability

The data obtained in this study were written in Japanese, and therefore, the raw data cannot be shared.
